# Advances in the application of gold nanoparticles in bone tissue engineering

**DOI:** 10.1186/s13036-020-00236-3

**Published:** 2020-05-06

**Authors:** Hongru Li, Su Pan, Peng Xia, Yuxin Chang, Chuan Fu, Weijian Kong, Ziyuan Yu, Kai Wang, Xiaoyu Yang, Zhiping Qi

**Affiliations:** Department of Orthopedic Surgery, The Second Hospital of Jilin University, Ziqiang Street No. 218, Changchun, TX 130041 PR China

**Keywords:** GNPs, scaffold, chemical modification, bone tissue engineering, Mesenchymal stem cells, delivery system

## Abstract

The materials used in bone tissue engineering (BTE) have been advancing with each passing day. With the continuous development of nanomedicine, gold nanoparticles (GNPs), which are easy to be synthesized and functionalized, have attracted increasing attention. Recent years have witnessed this amazing material, i.e., GNPs characterized with large surface area to volume ratio, biocompatibility, medical imaging property, hypotoxicity, translocation into the cells, high reactivity, and other properties, perform distinct functions in BTE. However, the low stability of GNPs in the biotic environment makes them in the requirements of modification or recombination before being used. After being combined with the advantages of other materials, the structures of GNPs have exhibited great potential in stem cells, scaffolds, delivery systems, medical imaging, and other aspects. This review will focus on the advances in the application of GNPs after modification or recombination with other materials to BTE.

## Introduction

At present, clinically, the gold standard treatment for bone defect is autologous bone grafts. However, autologous bone grafts are limited by defect shape, donor number, immunogenicity and other factors [1]. Hence the development of bone tissue engineering (BTE) turns out imperative. It is common knowledge that the key and difficulty in BTE is the formation of 3D scaffolds. To meet the needs of anatomical structure, functional recovery and even aesthetics, implanted bioactive scaffolds require every conceivable advantage. In addition to the requirements of mechanical properties, biocompatibility and controlled biodegradability, scaffolds also need to perform as adhesive carriers for cells and bioactive molecules (cytokines, inhibitors, drugs, antibiotics, and other molecules). Therefore, the capacity of scaffolds to promote cellular interaction, viability, and deposition of the extracellular matrix is also essential. Undoubtedly, inflammation and toxicity should also be minimized. In general, the purpose of BTE is to build a center of tissue regeneration and morphogenesis through such three-dimensional porous scaffolds [2–5]. In recent years, the use of nanoparticles, particularly metal nanoparticles, has expanded in BTE [6, 7]. GNPs have played as one of the most promising and valuable potential materials and tools. GNPs’ easy-to-control nanoscale size, easy preparation, high surface area, easy functionalization, excellent biocompatibility and other characteristics facilitate them to fulfill the relevant tasks in BTE [8]. Compared with other types of nanoparticles, GNPs’ low toxicity, colloidal stability, and outstanding physicochemical property which benefit from local plasmon resonance (LSPR) [9], have made them one of the key directions for the combination of tissue engineering and medicine.

**Fig. 1:**
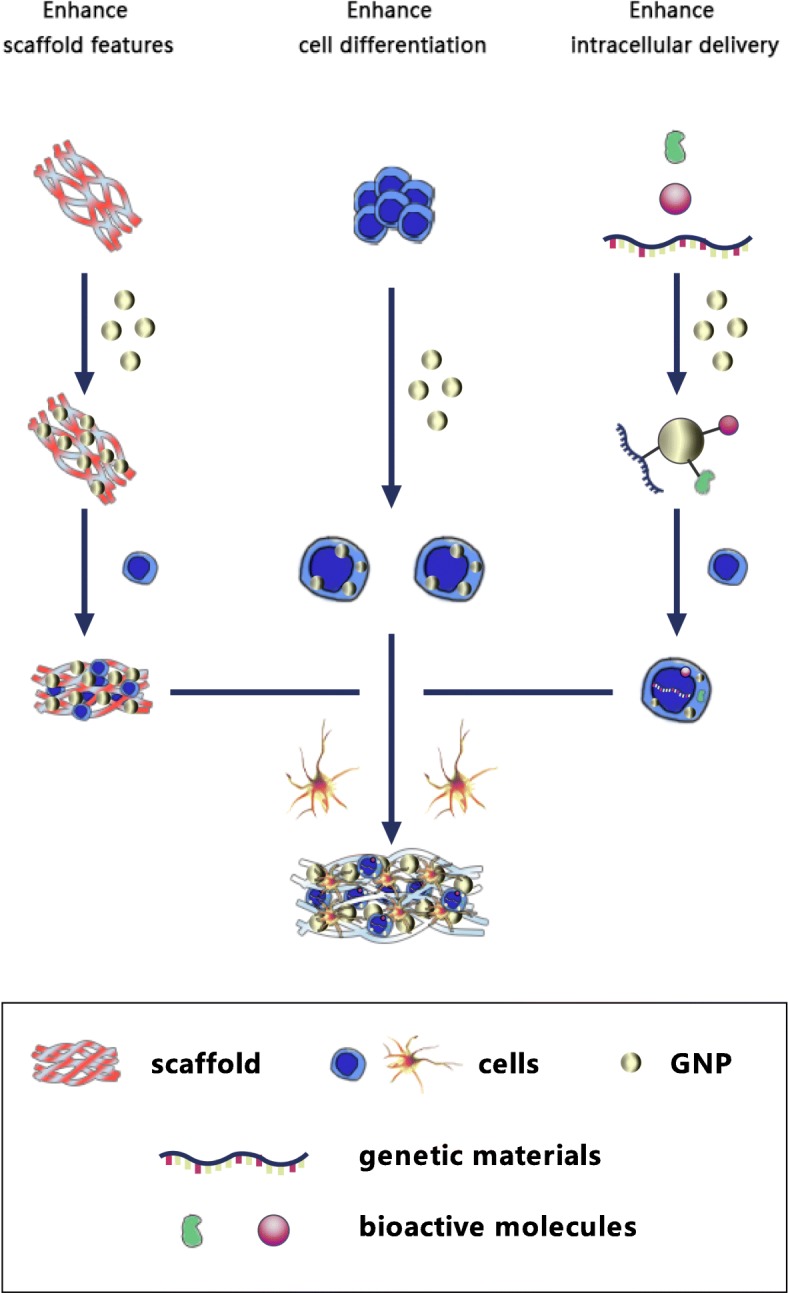
Schematic diagram of GNPs’ application to stem cells, scaffolds and intracellular delivery

Over the years, GNPs have been widely used for the preparations of biologic therapies, including delivery systems of drugs and genes, photographic developers, photothermal therapies, biosensors, diagnostic reagents, etc. [6, 9–12]. In recent years, the extensive presence in the three key elements of scaffolds, cells and bioactive molecules have indicated that GNPs have been widely used in BTE (Fig. 1). The present review aims to cover the main aspects around GNPs-containing biomaterials that are relevant to their applications to bone tissue engineering strategies. The review will start from the synthesis strategy to modification and recombination, that is, the way to regulate the properties of GNPs. Then, the review will focus on the application of GNPs to BTE, including some contributions oriented to clinical applications and commercial products. In the end, based on the complete explanation of the biological significance of GNPs in BTE mentioned above, the future research direction will be discussed and prospected, viewing to provide theoretical guidance for the next research trend. To the best of the authors’ knowledge, this is the first review paper dealing specifically with GNPs and related biomaterials in BTE.

## The synthesis, modification, and recombination of GNPs

### The synthesis of GNPs

The synthetic method of spherical GNPs initiated by Turkevish [13] in 1951 cannot be ignored; namely, HAuCl_4_ is treated with citric acid in boiling water, in which sodium citrate acts as both reducing agent and stabilizing agent. Later, G. FRENS [14] discovered that different proportions of citrate and HAuCl_4_ could be used to produce GNPs of controllable size, which is a historic method still in use today. The Seed-growth method [15] has been a mature and efficient synthetic method. However, the pivotal cetyltrimethylammonium bromide (CTAB) in the method, which forms a bilayer on the gold nanorod surface, is toxic [16]. Another method to large scale synthesis of GNPs reported in recent years is ultrasonic spray pyrolysis (USP) [17, 18], referring to that GNPs are extracted from gold (Au) filings in a thermal reactor. However, the process will be contaminated by other alloying. Therefore, green synthesis of GNPs in the field of biological application of GNPs was proposed. In addition to reducing cytotoxicity relatively, green synthesis, most importantly, is to control the size and shape of GNPs. Moreover, in the environment of biocompatible materials, GNPs’ stability and biological affinity can also be improved. Green synthesis of GNPs aims to achieve environmentally acceptable, economical, time-saving, and easily scaled up to large-scale synthesis. Past researches have shown that biological systems such as bacteria, fungi, actinomyces and plants are capable of producing GNPs [19, 20], and they are rich in different types of reducing and capping agents. Some of these are natural materials able to act as scaffolds for tissue engineering. When these elements are aggregated, the biological significance of the produced GNPs will be reinforced. For example, Sorina Suarasan and others [21] achieved one-pot, green synthesis of GNPs by taking advantage of gelatin biopolymer, in which gelatin performed as a unique reducing, growth controlling and stabilizing agent. The results showed that gelatin-coated GNPs enhanced the proliferation rate of osteoblasts with the increase of nanoparticle concentration. Similarly, gold nanodendrites and gold nanostars were successfully synthesized from natural gelatin biopolymers by using one-pot, green synthesis method recently, which have excellent GNPs biocompatibility in vitro [22]. Except for biocompatibility, green synthesis of CGA-GNPs produced by using chlorogenic acid as the bioactive reductants increased the anti-inflammatory ability [23]. GNPs/gelatin/LTF (lactoferrin) nanogels exhibiting enhancement of fluorescence (~ 50-fold) as compared to free LTF. GNPs/gelatin/HRP nanogels exhibited enhanced bioluminescence (~ 11-fold) in an HRP (horseradish peroxidase)-luminol system [24]. Concerning other biosynthesis methods, below reviews [19, 20] are strongly recommended. In brief, the use of bioactive reductants for green synthesis and their mechanism research will open up a new direction in biological nanomedicine.

### The modification and recombination of GNPs

The modification and recombination of GNPs prove crucial when applied to medical tissue engineering. Technologies of the modification and recombination of GNPs can maintain GNPs’ colloidal homeostasis, enhance their interaction with the biological environment, improve their biocompatibility, and extend their lifetime in the body. The accumulation of GNPs can better accomplish targeting, imaging, photothermal treatment, sensing, delivery and other objectives. The technologies of modification cover thiolation and polyethylene glycol (PEG) modification, amino acid modification, core-shell structure, electrostatic interaction, etc. The technologies of recombination include the recombination and crosslinking of GNPs and other biocompatible materials. It is generally believed that 3D biomaterials with comprehensive properties in tissue engineering (TE) are all produced by recombination. GNPs can play a more powerful function in such a structure by relying on other materials and their structural forms, to achieve the optimal compromise between the mechanical performance and biological characteristics in the whole system. Only by making the best of the various materials, can the complex objectives of TE be realized. Not only in BTE, modified and recombined GNPs have ample scope for skin [25], nerve [26] and myocardial tissues [27].

#### Thiolation and polyethylene glycol (PEG) modification

Mercaptan with strong affinity provides relatively stable S-Au bonds when reacting with Au. Therefore, thiolation is one of the important means of surface modification of GNPs. Often used as GNPs coating, PEG in the delivery system extends the drug cycle time, improves the solubility of hydrophobic drugs, and minimizes the non-specific uptake [5]. This anchoring can be achieved by thiolated PEG, where one end of the GNPs is functionalized by hydrosulphonyl and the other end by other functional groups, namely ligand exchange [5, 28]. The effects of GNPs’ size, PEG molecular weight and NP - terminated ligands on graft rate and graft density, as well as the distribution in internal organs, toxicity, are being investigated [29–31]. Haram Nah et al. [32] used thiol-PEG-vitamin D (SPVD) to conjugate vitamin D to GNPs and found that produced VGNP could not only effectively enhance osteogenic differentiation of human adipose-derived stem cells (human adipose adipose-derived stem cell are abbreviated hADSCs), but also be used as a new functional carrier in BTE.

#### The modification of amino acids

The modification of amino acids can also be described as the modification of peptide or protein. GNPs can easily achieve functionalization with thiols, amines, or even phosphine moieties of biological molecules, particularly, thiol-containing molecules mentioned above. As a result, new hybrid materials of amino acids, peptides, proteins and GNPs are also produced [33]. The main methods for making such functionalized GNPs are ligand exchange, chemical reduction, and chemical conjugation. For an in-depth understanding of amino acid /GNPs components, Sandhya [34] found that the interaction trend between amine terminal and Au was the highest by using density functional theory (DFT), which was confirmed by Natural Bond Orbital Analysis (NBO). Molecular dynamics simulation [35] also analyzed and compared the geometric shapes and flexibility of 20 adsorbed amino acids. The results found that Asp on the surface of GNPs adsorbed faster than other amino acids, whilst Asp plus Ser, Arg and Thr presented higher flexibility on GNPs than other amino acids. Pro, Cys, and Met showed more stability on the surface of GNPs, especially on 8 nm GNPs. In the link with peptide, the presence of GNPs did change the structure and dynamics of the peptide, and the extent of the effect depended on the sequence of the peptide. Conjugated peptides usually present reduced conformational flexibility and the amount of reduction depending on the amino acid sequence [36]. According to Fatemeh [37], compared with isotype dipeptide and monoamino-acid, when adsorbed on the gold (Au) surface, isotype tripeptide presented more flexibility, more gyration, and the farthest distance from the GNPs. This suggested that peptides should be designed with appropriate secondary structures in the delivery system. GNPs functionalized by peptides, which are also named capping agents, avoid aggregation in ionic solutions. The whole system terminated by peptides will have pH responsiveness, self-assembly, and enhanced biological stability, all of which facilitate the delivery and controlled release [38–40]. GNPs modified by peptides can improve cellular uptake, nuclear transport, and intracellul [41]. Based on the latest studies of the peptide-functionalized gold nanoparticles (peptide-GNPs) on delivering drugs, siRNA, imaging, etc. [39, 42–44], there are sufficient reasons to look forward to their prospective delivery and medical imaging applications in the treatment of osteosarcoma and BTE.

#### Core-shell structure

Core-shell structure primarily refers to the inorganics’ or polymers’ coating, embedding and encapsulation on GNPs. Core-shell structure as high function materials characterized by modification has received increasing attention. Such bi- concomitant system can improve the performance of nuclear particles like reducing reactivity or changing thermal stability to enhance the stability and dispersion of nuclear particles. The system displayed the unique characteristics of all applied materials, which could manipulate the surface functions to meet various application requirements [45]. Regarding the inorganic shell for GNPs, silica has received more focuses. As early as 1996, Nanosized Gold−Silica Core−Shell Particles were prepared [46]. The system has high reactivity, in which silica can also act as a stabilizer and show excellent optical properties. With the development of the research, a mesoporous silica-gold nanorods system has been developed, which is a multilayered structure by layer-by-layer assembly with rotational diffusion and adjustable size [47]. When assembled with proteins, the system with high porosity and biocompatibility can function as both the targeted drug carrier and medical imaging contrast agent [48]. What’s even more exciting is that the surface encapsulation of silica gel shell could inhibit the cytotoxicity of GNPs used in cells [49].

**Fig. 2:**
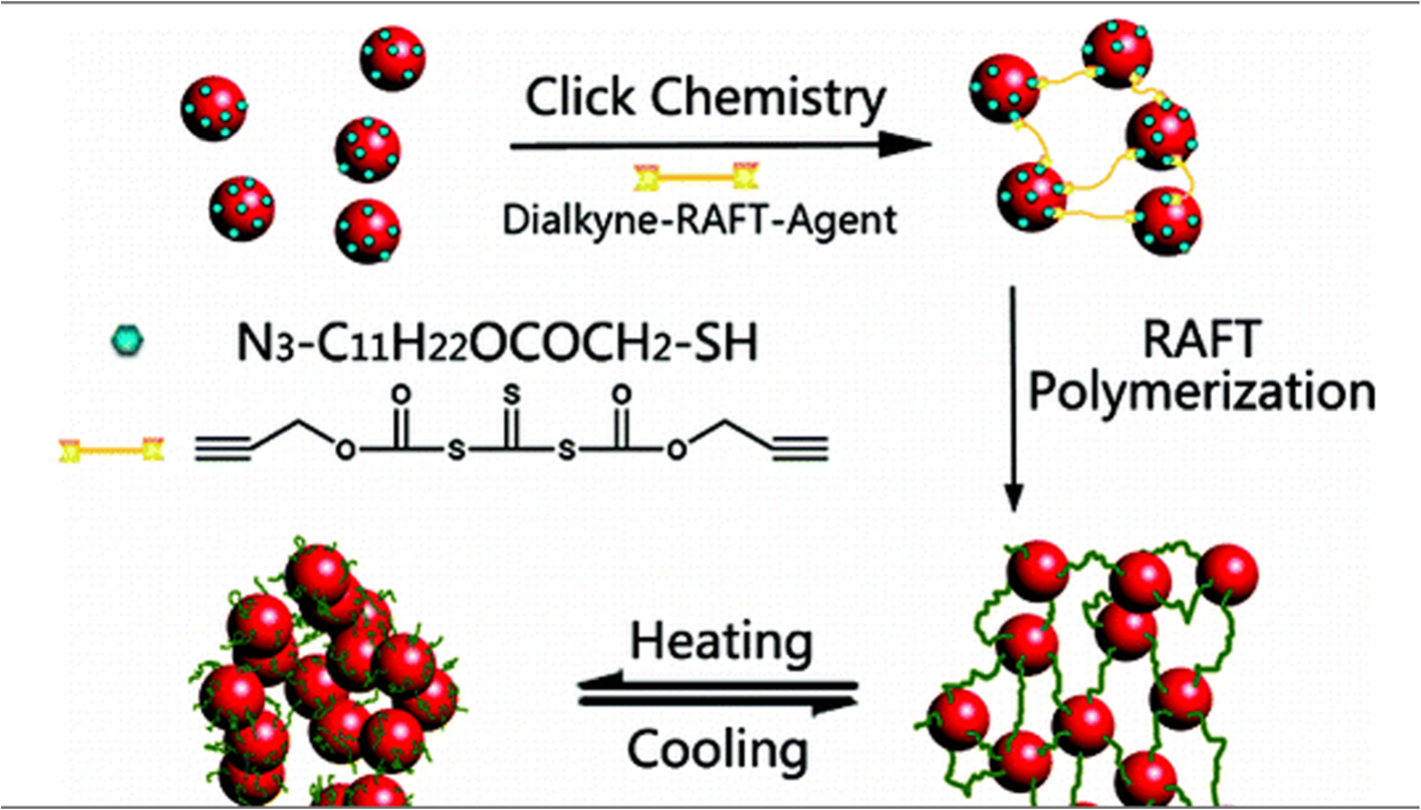
The schematic diagram for the polymerization preparation of thermosensitive nano-hydrogels based on click reactions and reversible addition−fragmentation chain-transfer. Reprinted with permission from Lian, X. et al. [57] Copyright© 2010, American Chemical Society

As for polymers, Polyaniline Coated Gold Nanorods (PANI-GNRs) had been yielded by chemical polymerization in situ [50], which showed both bactericidal and redox activities. Au, PANI nucleus and shell nanocomposite [51, 52] also have unique biological responsiveness due to their unique optical and electrical properties. Other noteworthy types of polymer are intelligent polymers such as Poly (N-isopropylacrylamide) (PNIPAM), which show performance changes in environmental conditions of temperature, pH, light, etc. After the formation of the core-shell structure, the corresponding biological behavior of PNIPAM and other polymers can be combined with the optical properties of GNPs, which is conducive to the further expression of better overall material properties. Early, Jiang et al. [53] took advantage of simple electrostatic adsorption to form Poly(N-isopropyl acrylamide) (PNIPAM) microspheres as cores and gold as shells. GNPs were combined with PNIPAM by electrostatic adsorption, which could be demonstrated by UV-Vis, TEM, and FT-IR with promising drug release applications. However, recent researches have been apt to PNIPAM microspheres as shells and gold as cores [54–56]. The production methods cover click reactions and laser heating resulting in the aggregation of GNPs, also called “grafting-from”, and the “grafting-to” approach. The first scenario was GNPs decorated with azide groups (GNPs-N3) which were prepared through ligand exchange. Click reactions between GNPs-N3 and dialkynetrithiocarbonate yielded cross-linked GNP aggregates [57] (Fig. 2), which were ideal of tolerance, high absorption and high selectivity to many functional groups. The second could lead to poor polymer size control. Double-responsive hydrogels based on thermosensitive PNIPAM and poly(N,N-dimethylaminoethylacrylamide) (PDMAEMA), pH [58], PNIPAM and redox-responsive poly (ferrocenylsilane) (PFS) and redox was prepared, which broader corresponded to the complex responses in living nature, and also could act as the reducing agent to prepare GNPs in situ in the hydrogel. Polymers were by the combination of single-electron-transfer living radical polymerization, atom-transfer radical polymerization, and click chemistry. Such materials have great potential to become intelligent in-situ injectable and tissue-engineering scaffolds.

#### Electrostatic interactions

**Fig. 3:**
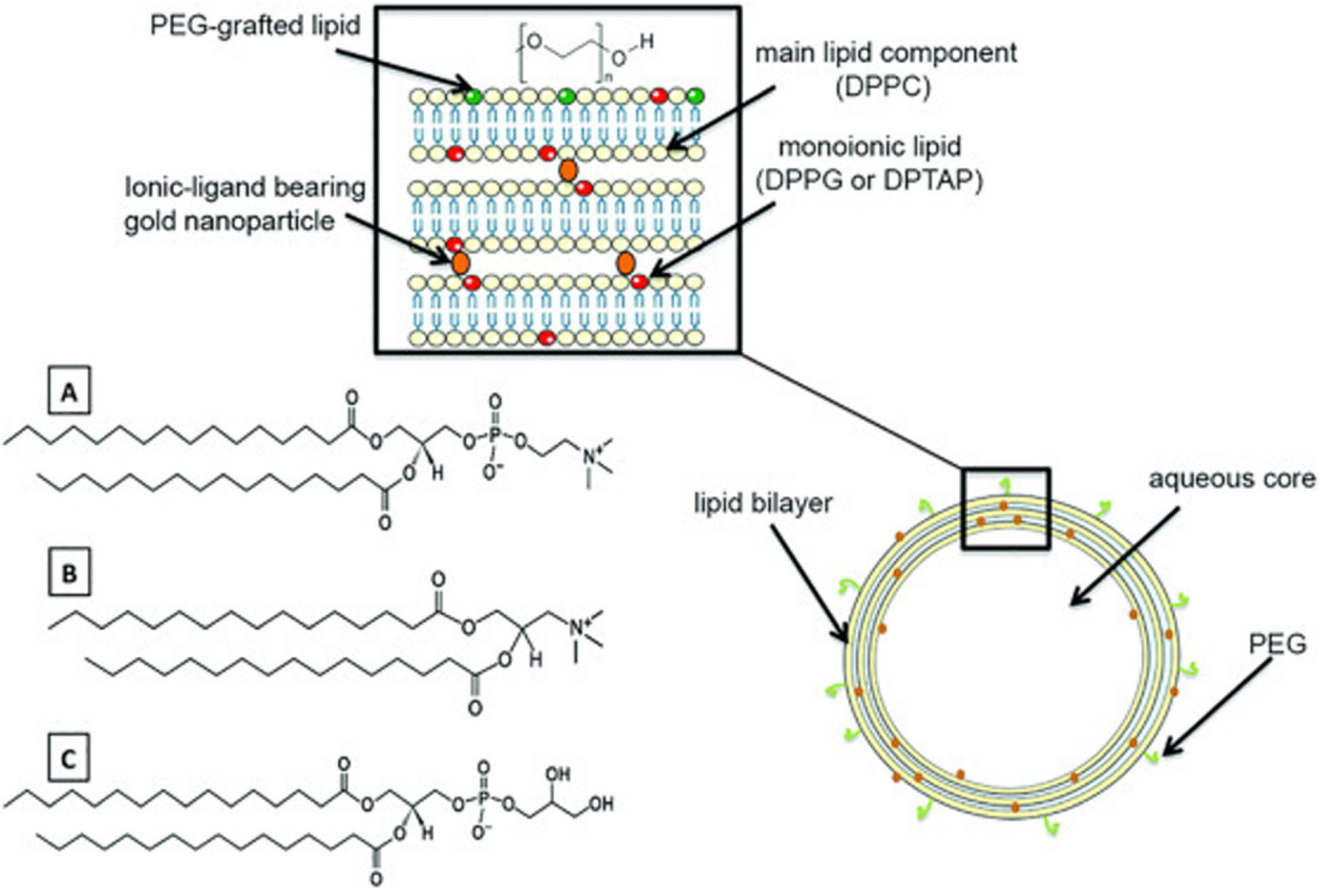
Proposed structure of lipo-GNPs with the underpinning lipid-nanoparticle interaction at the aqueous-bilayer interface highlighted (insert). Lipid structures utilized in the preparation of NCL-PCG and PCL-NCG are presented **a**: DPPC, **b**: DPTAP, **c**: DPPG. Reprinted from Elbakry, A. et al. [59] Copyright 2017, with permission from Elsevier

**Fig. 4:**
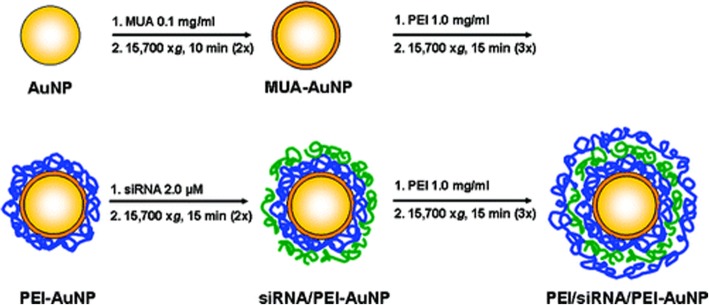
Flowchart Illustrating the LbL Deposition Applied to GNPs. Reprinted by permission from Elbakry, A. et al. [61]. Copyright (2009) American Chemical Society

**Fig. 5:**
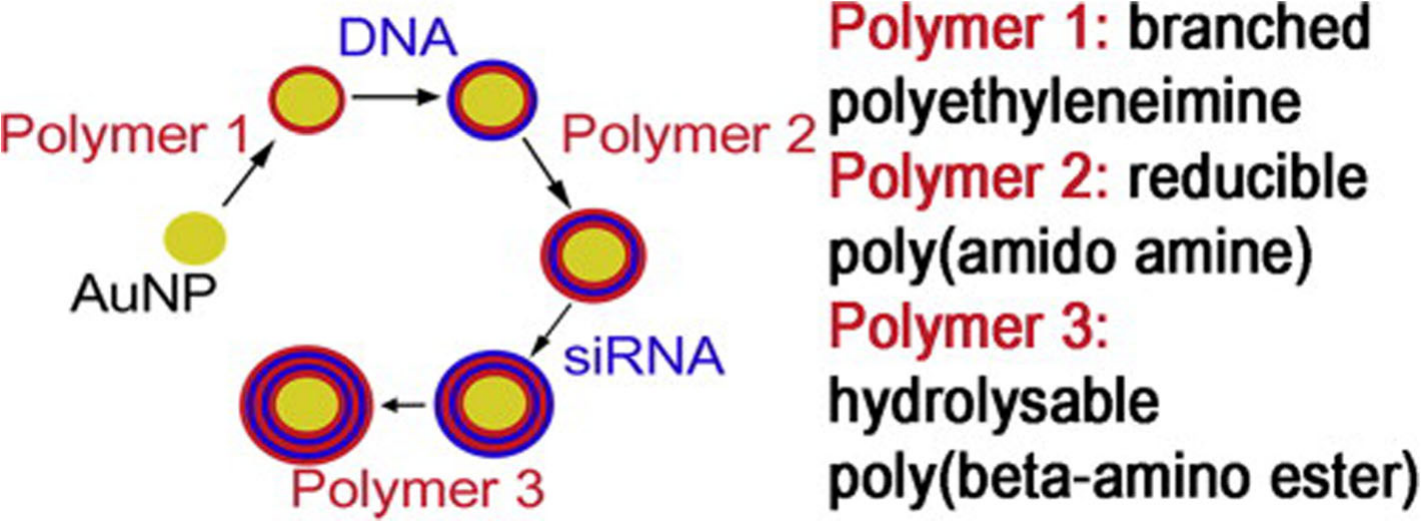
LbL process starting with Mau [63]. Copyright 2015, with permission from Elsevier

Electrostatic interaction is one of the main methods of non-covalent surface modification for GNPs. Gennaro et al. [59] prepared the liposome-gold nanoparticles (lipo-GNPs) system (Fig. 3) by reversed-phase evaporation, in which the cationic or anionic surface-functionalized particles were bound with the lipid composition with opposite charges, followed by the modification of pegylation. Lipo-GNPs protected, preserved and isolated the internal substances to improve cell uptake, while external liposomes provided further modification platforms, which were beneficial for achieving the unity of multi-functional and multi-therapeutic components. Layer-by-Layer (LBL) is also a research hotspot taking advantage of electrostatic interaction modification. Biodegradable polymers with positive and negative charges can be deposited and adsorbed on GNPs layer by layer, and then combined with small molecular genetic material by electrostatic binding, which can be used as effective carriers for delivering DNA, siRNA, and microRNA. These delivered small molecules can act on protein targets to reduce cell proliferation [60, 61], and induce the differentiation lineage of stem cells [62]. After being internalized by cells, they are beneficial to realize the next function [63], which has the potential of applications to bone tissue remodeling in vivo. The partial synthesis process and materials are shown in Figs. 4 and 5.

#### Recombination

To meet the complicated requirements of cell, tissue and organ, a variety of materials in the artificial structure of BTE require modification and recombination. CNPs are no exception. A large number of reports have expounded that GNPs are mixed and combined with hydrogels, bioactive glass, electrospinning materials,3D printing materials, etc. to achieve TE treatment goals [10].

Hydrogels have highly hydrated and interconnected structures, which can be formed in situ after injection. Besides, hydrogels with simple preparation, low toxicity, can also be applied to the responsiveness of the environment [64, 65]. Bioactive glass can induce bone matrix formation and enhance mineralization [66]. Gold-containing bioactive glasses is a solid-state synthesis to produce alternative biomaterials for bone implantation. Electrospinning materials have high tensile strength, adjustable porosity and excellent electrophysiological performance [67, 68]. 3D printing materials can refine the implant and enhance the information exchange with cells [69]. GNPs recombined with different types of materials focus on different functions, due to many possibilities provided by the applications of the diversity of material types and structural forms to BTE.

Composite materials fall into two categories, namely natural compounds and synthetic compounds. Natural compounds include chitosan, gelatin, collagen, etc., characterized with high biocompatibility, biodegradability, low immunogenicity, low toxicity, etc. but usually poor mechanical modulus [70]. While Synthetic compounds such as Polylactic acid (abbreviated PLA) and poly (D, l) -lactide-glycolide copolymer (abbreviated PLGA) are considered to have adjustable mechanical properties. However, poor biocompatibility of synthetic compounds can lead to poor integration with bone tissue [71]. In conclusion, according to their advantages and disadvantages combined with GNPs, there are different applications in BTE worth developing, which are not limited to making scaffolds. These will be covered in detail in section 3 below.

## The application of GNPs in BTE

### Optimizing stem cells

BTE aims to optimize and enhance the osteoblast differentiation and matrix mineralization of stem cells and other cell types dominated by osteoblastic precursor cell lines, with a basic standard of a high level of alkaline phosphatase and mineralization, gene expressions Runx2, BMP-2, and OCN [72, 73]. Mesenchymal stem cells (MSCs) are considered the progenitor cells of bone tissue and are known to differentiate along multiple lineages, including osteoblasts, chondrocytes, and adipocytes [74]. Regarding the mechanism of GNPs inducing osteogenic differentiation, Yi et al. [75] believed that GNPs could interact with proteins in the cytoplasm and the extracellular matrix through mechanical stress, causing activation of the p38 MAPK signaling pathway, which in turn causes up-regulation of osteogenic genes and down-regulation of adipogenic genes. Otherwise,Zhang et al. [76] suggested the activation of the ERK/ MAPK pathway is involved in differentiation and mineralization of primary osteoblasts,and GNPs increased the level of ERK phosphorylation/total ERK. 20-nm GNPs are more effective than 40-nm GNPs. Activation of this pathway can also improve the adherence and proliferation of myoblasts and enhance skeletal muscle repair and regeneration [77]. GNPs, on the other hand, can act as antioxidants in bone marrow-derived macrophages [78], reduced the production of reactive oxygen species in response to RANKL (nuclear factor-κB ligand) and upregulated RANKL-induced glutathione peroxidase-1 (Gpx-1),affecting osteoclast formation [79], and then preventing bone resorption. However, regarding the ability of nanostructures inducing osteogenic differentiation, Mahmoud et al. [79] compared Hydroxyapatite (HA-NPs), Gold (GNPs), Chitosan (C-NPs), Gold/hydroxyapatite (G/HA-NPs) and Chitosan/hydroxyapatite (CH-NPs) on bone marrow-derived mesenchymal stem cells (BM-MSCs). Among them, GNPs and G / HA-NPs have the most significant effect. This ability is also related to the concentration, size and shape of the GNPs [80, 81]. Ko et al. [82] found that GNPs enhanced bone differentiation of adipose-derived stem cells (ADSCs) at the sizes of 30 nm and 50 nm. By comparing several shapes and sizes of GNPs, Li et al. [83] found that the size and shape of GNPs affected the osteogenic differentiation of hMSCs by regulating the activation of Yes-associated protein (YAP). In the research of superparamagnetic iron oxide (SPIO)-Au core-shell nanoparticles for promoting osteogenic differentiation of MC3T3-E1 cells, dependences existed on the concentration of the GNPs system [84]. Since the application of GNPs to BTE is at the initial stage, generally, there is a lack of more detailed studies in this area. Until recently, Dong et al. [85] demonstrated that the fate of human mesenchymal stem cells (hMSCs) could be regulated by the inherent physical cue of the material surface down to atomic-scale features. By revelation, Au nanowire-patterned array platforms with multiscale design from the macroscale to the nanoscale were developed for studying human bone marrow-derived mesenchymal stem cell (hBM-MSC) response [86], when the angle of the Au nanowires on glass was increased from 0° to 90°, hBM-MSC arrangement exhibited a transition from a unidirectional distribution induced by a vector response to a bimodal polarization pattern. The results indicated that the nanoscale control of cellular tissue was closely related to the micro-nano morphology of the material surface. Soft-lithographic design provided new models and insights into the deeper understanding of cell-nano-biointerface interactions.

People are certainly not satisfied with the application of just GNPs. Studies [87] on hMSC cultured by GNPs with different charges after modification found that positive and neutral charges had no abnormal effect on cell behavior and were highly absorbed by cells, while treatment of GNPs-COOH with negative charge reduced ALP activity (the activity of alkaline phosphatase) and matrix mineralization in hMSC. GNPs–COOH-treated hMSCs gene expression profiles suggested that up-regulation of growth factors FGF-2 and TGF-β genes can promote cell proliferation and inhibit ECM development,which indicated the usefulness in promoting re-aggregation of osteoblast density at the injury site. Li et al. [81] prepared arginine–glycine–aspartate (RGD)-modified gold nanoparticles (GNPs) with tunable surface ligand density to mimic the ECM microenvironment. The experimental results showed that the biomimetic GNPs regulated the osteogenic and adipogenic differentiation of MSCs mainly through affecting the focal adhesion and cytoskeleton.

**Fig. 6:**
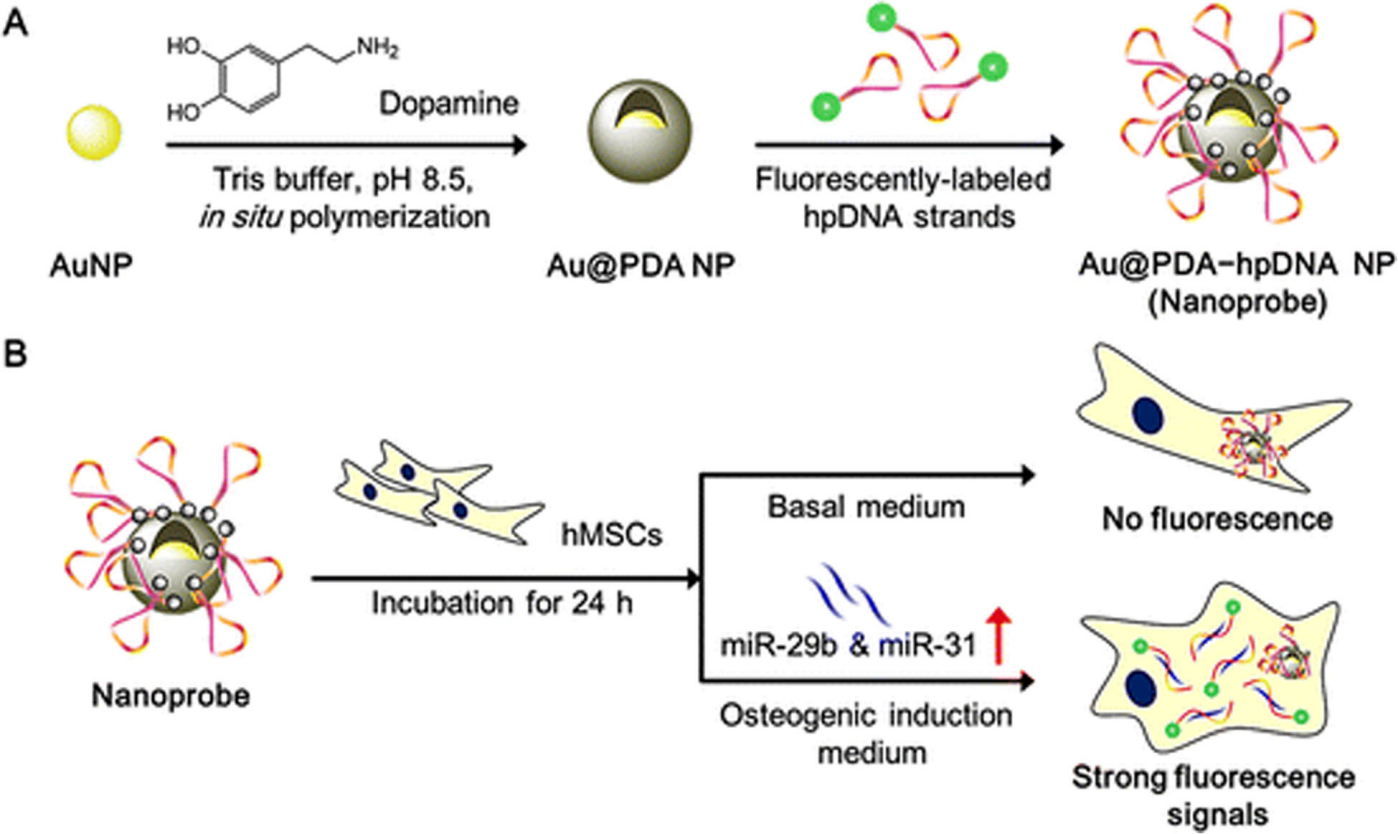
(**a**) Preparation of the Polydopamine-Coated GNPs (Au @ PDA NPs) and Hairpin-DNA-Based (hpDNA) Nanoprobes; (**b**) Intracellular Detection of miRNAs in Living Human Mesenchymal Stem Cells (hMSCs) Reprinted with permission from [88] . Copyright (2015) American Chemical Society

Nor is the association between GNPs and BTE stem cells limited to enhanced osteogenic differentiation. Interestingly, Choi et al. [88] developed a nanoprobe that could monitor the differentiation of stem cells over time, based on polydopamine-coated GNPs. The PDA shell facilitates the immobilization of fluorescently labeled hairpin DNA strands (hpDNAs) that can recognize specific miRNA targets. They labeled hairpin DNA strands (hpDNAs) onto the PDA shell simply by π–π interactions (Fig. 6). This technique has broad prospects in the study of stem cell differentiation kinetics, identification and isolation of specific cell types, and high-throughput drug screening. This is also the surprise of GNPs.

### Enhancing the performance of scaffolds

The complexity of bone structure and the high destructiveness of bone injury lead to the difficulty of bone healing. BTE scaffold is developing towards multi-combination and multi-functional biomaterials. We encourage that scaffolds to mimic the extracellular matrix (ECM) to communicate more with the biological microenvironment to guide and enhance osteogenesis and mineralization, and even the ability to form blood vessels [9, 89, 90]. Composite natural or synthetic polymer scaffolds incorporating the characteristics of GNPs and other tissue-engineered materials have also been manufacturing in combination with both natural and synthetic polymers. Here, GNPs have been targeted for promoting bone cell proliferation and differentiation, mechanical strength, electrophysiological signaling, and antioxidant activity.

#### Composite natural polymer

Natural polymers are characterized with high stability, low toxicity, non-thrombogenic and non-immunogenic properties, in which fibrin, collagen, hyaluronic acid, etc. are the main components of ECM. However, natural polymers also have problems such as low mechanical modulus, complex structure, high production cost, large differences between batches, low biological adhesion potential, difficulty to be controlled by in vivo enzymes, and so on [91, 92]. When combined with GNPs, they will produce more or less effects making the best use of the advantages and bypassing the disadvantages (Table 1). Hydrogels composed of three-dimensional hydrophilic polymer chains are a common form of potential materials, with characters of controlled porosity, encapsulation, absorbability, and injectability [71, 108]. Based on the method of LBL (layer-by-layer self-assembly), Vieira et al. [93] prepared 7 nm gellan gum (GG)hydrogel-like shell around individual AuNRs, which contained poly (acrylic acid) with positive and negative electric charges, poly (allylamine hydrochloride) and GG. Cell compatibility and osteogenesis of GG-coated AuNRs had also been resolved. The polysaccharide coating also prevents GNPs from aggregation. Besides, Au is distributed along collagen fibers within the matrix of type II collagen--Au nanocomposites [94], and the nanocomposites promoted the proliferation of cartilage cells and up-regulated certain genes. These enhancements may be related to the appropriate characteristic size, greater modulus, and greater antioxidant activity of the nanometer features on the surface. Photo-curable gelatin hydrogels (Gel) [95] can also cross-link GNPs. The hydrogel carrying GNPs accelerate the osteoblast differentiation of ADSCs in a dose-dependent manner, thereby promoting proliferation, differentiation and ALP activity. Pourjavadi et al. [97] created an injectable hydrogel that was synthesized by chitosan, κ-carrageenan, and poly (NIPAM). Gold nanoparticles were used to enhance MG-63 cell growth and adhesion, which were improved greatly. Such materials are both conductive and injectable. Also, the Gel-Ty hydrogels containing G-NAC (Gel-Ty/G-NAC) prepared by Lee et al. [96] in 2018 is injectable, with the main body of GEL and GNPs to promote the growth and osteogenesis of hADSCs. Thermo-sensitive hydrogel based on chitosan/pectin/GNPs (CS/Pec/GNPs) has also been successfully prepared [98]. The gelatinization temperature varies when the concentration of pectin and GNPs are different. The hydrogel has the right pore size and high cellular compatibility, which has been proven to be an irritant for bone remodeling (Mc3t3-e1). Good precedents have shown that similar scaffolds are useful in heart tissue engineering [109]. Ribeiro et al. [99] used silk fibroin as the matrix and the reducing agent to make antibacterial SF / nanohydroxyapatite hydrogels with silver and gold nanoparticles. After the in situ synthesis of silver and gold nanoparticles, the original antibacterial properties and bone conductivity of the system were improved. 3D bio-printed hydrogel includes automatic deposition of cells and biomaterials to mimic the structure of tissues. GNPs serve as dynamic multivalent cross-linking agents for thiol-modified HA (CMHA-S) and thiol-modified gelatin (Gtn-DTPH) [100], which prevent the static cross-linked gel structure from being broken and blocking the printer halfway. The resulting synthetic extracellular matrix biology can be printed into tubular structures that support cell proliferation and matrix remodeling. In addition to the pre-osteoblasts mentioned above, the osteogenic differentiation of human periodontal cells (hPDLCs) was also significantly enhanced by GNPs modified by human β-defensin 3 (hBD3), and by activating the Wnt / β-catenin signaling pathway in the inflammatory microenvironment [101]. GNPs in injectable calcium phosphate cement (CPC) enhance osteogenic differentiation of human dental pulp stem cells,including better wetting, greater protein adsorption, and improved cell attachment and spreading [102]. In addition, GNPs composite natural polymers are also found in soft tissue engineering materials. For instance, crosslinking acellular porcine tendon has been used to improve the enzymatic hydrolysis resistance and biocompatibility [110].

**Table 1:** Composite natural polymer

Composite material	Cells used	Enhanced performance	References
GG	SaOS-2	Cell compatibility and osteogenesis; preventing GNPs from aggregation	[93]
type II collagen	Chondrocyte	Appropriate characteristic size, larger modulus and greater antioxidant activity; promoting proliferation	[94]
Gel	ADSCs	Proliferation, differentiation and ALP activity, cell growth, contributing to bone, injectability	[95, 96]
chitosan, κ-carrageenan,	MG-63	Conductivity and injectability	[97]
CS / Pec	MC3T3-E1	Thermosensitivity; appropriate aperture; high cell compatibility	[98]
SF	osteoblastic cells	Antibacterial property; bone conductivity	[99]
HA/Gtn	osteoblastic cells	3D printing; dynamic crosslinking agent	[100]
hBD3	hPDLCs	Osteogenic differentiation	[101]
CPC	hDPSCs	Better wetting; greater protein adsorption, and improving cell adhesion and spreading	[102]
hydroxyapatite (HAp) ,Ag	MG-63	Antibacterial; non-toxic; inducing bio-mineralization	[103–105]
HA/Graphene	osteoblastic cells	Catalyst; excellent 3D structure; bone conductivity	[106, 107]

#### Composite synthetic polymer

Synthetic polymers include poly lactic-co-glycolic acid (PLGA),poly (ethylene glycol) (PEG), poly (vinyl alcohol) (PVA), poly (2-hydroxyethyl methacrylate) (PHEMA), polyacrylamide (PAM), etc. [111] Mature processing technology, excellent mechanical properties, good environmental sensitivity, and electrophysiology are their advantages, but lack of biocompatibility [112]. In addition to the intelligently responsive hydrogels mentioned in **2.2.3**, nanofibrous composites with thiol-end groups were prepared using electrospun PLGA [113]. GNPs can be used as a bone inducer of ADSCs in vitro with good dispersion and adjustable concentration onto them. Terranova et al. [114] introduced porous polystyrene (PS) microfibers prepared by grafting GNPs surface-functionalized with mercaptosuccinic acid,which improved bone reconstruction in a model of critical size calvarial defect in mice with β-Tricalcium phosphate(β-TCP). Polypropylene fumarate (PPF): diethyl fumarate (DEF) (7,3 w/w) biodegradable photocurable polymers with incorporated GNPs [115] and electrospun poly(L-lactide) and gold nanoparticle composite scaffolds [116] can be applied to musculoskeletal tissue engineering, all of which are representative of the conductive, biodegradable scaffolds incorporated into GNPs (Table 2).

**Table 2:** Composite synthetic polymer

Composite material	Cells used	Enhanced performance	References
PLGA	ADSCs	bone inducer,good dispersion and adjustable concentration onto cells	[113]
PS,β-TCP	human mesenchymal stem cells (hMSCs)	cytocompatibility, biocompatibility	[114]
PPF,DEF	adipose stem cells (ASCs)	induced muscle tissue regeneration	[115]
Poly(L-lactic acid) (PLLA)	Muscle cells	biodegradable, biocompatible and conductive scaffold for skeletal muscle repair	[116]

### Delivery systems

In addition to enhancing 3D scaffolds, another attractive advantage of using GNPs in BTE is to form the delivery system. GNPs’ large surface-to-volume ratio, ability to penetrate cell membranes, ease of functionalization and targeting [6, 12], and its ability to promote wound healing and angiogenesis [117] make them a powerful tool for delivery systems. That is, in addition to growth factors, drugs, and genetic material, it also has the value of being delivered.

Bioactive glass containing GNPs has been prepared as an alternative biomaterial for bone implants. Molten bioactive system [66] made by sol-gell derived apatite—bioactive glass composites [118] and exploiting a post-synthesis thermal treatment can produce hydroxyapatite while controlling release GNPs, which not only rebuild bone tissue but also have antibacterial properties. This well-validated model for controlled delivery of GNPs can further functionalize GNPs to improve material function. Core-shell structure mentioned in **2.2.4**, and LBL (layer-by-layer self-assembly) and Lipo mentioned in **2.2.3** are also very good delivery models, which have been applied to delivering genetic material to induce bone differentiation and therapeutic gene expression [62, 119, 120]. Similarly, such applications also exist in bone - integrated dental implants. First, Ti-GNPs significantly enhanced osteogenic differentiation by increasing mRNA expression of osteogenic differentiation-specific genes in ADSCs. First, Ti-GNPs significantly enhanced osteogenic differentiation by increasing mRNA expression of osteogenic differentiation-specific genes in ADSCs. Ti implanted with GNPs showed a significant increase of bone interface [121]. On this basis, we used chitosan gold nanoparticles (Ch-GNPs) to couple DNA plasmids, causing osteogenesis advantageous, which are conducive to gene expression, and promote bone integration and formation [122, 123]. Also, the application of half-shell nanoparticles responsive magnetically [124, 125] and GNPs systems that carry drugs and active molecules and release alginate hydrogels [126] by ultrasound, have achieved preliminary results in respective bone-related therapeutic areas. Once delivered to cells, through precise environmental changes to determine the release of delivery molecules are also a future trend.

### Medical imaging

GNPs’ medical imaging capability [127] is useful for both stem cells and scaffolds. The aim is to continuously monitor stem cells in vivo, promote stem cell behavior and the great potential of new blood vessel formation [128], and track the accuracy, number and redistribution of stem cell differentiation and tissue repair. The scaffolds are for longitudinal monitoring, tracking the degradation of the material and identifying the ingrowth of new bone. The application to stem cell imaging first requires an accumulation of a certain number of GNPs in the cell.

In 2018, Narayanan et al. [129] demonstrated for the first time the ability of BM-MSC bio-reduced Au (III) while produced biocompatible GNPs in cytoplasm and nucleus. When he doped BM-MSC with 1 mM gold chloride in phosphate buffer saline at pH 7.4 for 28 days, the growth of BM-MSC to approximate 90% degree of fusion showed intracellular GNPs accumulation. Their research provided the theoretical basis and prerequisites for the subsequent application in BTE. The previous studies are MSC. For example, GNR coated with silicon dioxide (SiGNR) served as a photoacoustic imaging contrast agent (PA) to mark MSC [130]; cells can be monitored and tracked using US/PA imaging by labeling MSC with gold nanometer tracer (AuNTs) and altering the optical properties of MSC [131]. However, the shortcomings of light scattering, inaccurate reconstruction and difficult detection of deeper tissues have not been solved in the above researches on PA.

Based on the high X-ray attenuation of GNPs, Celikkin et al. [132] scanned the 3D-printed gelatin methacrylate (GelMA) and GelMA-GNPs scaffolds for bone defects using CT to demonstrate that GelMA-GNPs is a good solution for bone tissue engineering with enhanced imaging visibility of μCT imaging. In another classic study, labeled CPC with perfluoro-15-crown-5-ether-loaded (PFCE) poly (latic-co-glycolic acid) nanoparticles (hydrodynamic radius 100 nm) and GNPs (diameter 40 nm), as 19F MRI (Magnetic Resonance Imaging) and CT contrast agents, were proved that they could enhance the image contrast and accurately identify and locate the scaffold [133].

We have to think about what the real meaning is in clinical application. Bone-filling of periodontal disease, rarefaction of bone, diabetes, etc. and diseases with long-term follow-up or revision programs are suitable for such needs. After the selection of appropriate biological agents, pharmacokinetics, toxicology, subsequent excretion and other issues also need to be comprehensive and lengthy evaluation [134].

## Challenges

Biosecurity is one of the most important considerations in tissue engineering. The 3D material system shall be bioinert or biodegradable. The biosafety and biocompatibility of GNPs depend on size, volume, concentration, shape, cell uptake level, etc. [135–137] At the same time, toxic residues should focus on when chemical reactions are involved in each step from the chemical synthesis, modification to recombination with other materials. In addition, the intracellular response of GNPs still needs further study. Previous studies have shown that GNPs can cause mitochondrial membrane destruction and cell content loss, which is related to the production of reactive oxygen species (ROS) [138] Recently, antioxidant conjugation has been used to remedy this problem [139] The study of complex cytological effects of materials is also urgently needed. For example, cell damage and apoptosis caused by PEG-coated GNPs can be inhibited by antibodies formed after peptide functionalization [140]. The lack of systematic and detailed evaluation and detection criteria for cytotoxicity poses challenges for the further application of GNPs. The effects of these substances on the environment also need to be considered. Also, inapposite cell adhesion and intercellular interactions shall be solved in the patterning of GNPs - based nanosurface topography. More detailed cell arrangement and the addition of peptides may be improved. Finally, the timeless challenge in BTE is how to form the perfect biomimetic substrate, and how much GNPs can affect in BTE, which will take a long time to test.

## Discussion

The application of GNPs in BTE is new in the initial stages of development, with no prospect of weakening. Facing numerous challenges in BTE, it is necessary to find standard regimens able to meet the requirements and bear monitoring of time. We argue that large scale manifestation and comparative trials are needed to assess the cytotoxicity and subsequent ecological cycles. The methods and procedures to develop toxicity, efficacy, and clinical safety tests still need to be standardized. Currently, we believe that after the green synthesis of GNPs, the selection of more detailed size and shape to adapt, the combination of multi-material and multi-function, the combination with soft lithography and patterning, and the completion of more detailed communication between cells are the future trend. More challenging structures, such as intermolecular self-assembly hydrogels [141], have been prepared. Undoubtedly, GNPs prove one of the most promising BTE tools. Despite the previous studies, the bio-responsive GNPs system still needs continuous research and design. In vivo applications, in particular, a great deal of research is needed to provide experimental data. They shall benefit humankind on the premise of safety and reliability. It is believed that GNPs able to reconstruct the organizational environment will play an important role in the eventual translation to clinical applications.

## Data Availability

Not applicable.
